# From Inert Storage to Biological Activity—In Search of Identity for Oxidized Cholesteryl Esters

**DOI:** 10.3389/fendo.2020.602252

**Published:** 2020-11-20

**Authors:** Ayelet Gonen, Yury I. Miller

**Affiliations:** Department of Medicine, University of California, San Diego, San Diego, CA, United States

**Keywords:** cholesteryl ester, oxidized, macrophage, atherosclerosis, cardiovascular disease, biomarker, inflammation, toll-like receptor 4

## Abstract

Esterification of cholesterol is a universal mechanism to store and transport large quantities of cholesterol between organs and tissues and to avoid toxicity of the excess of cellular cholesterol. Intended for transport and storage and thus to be inert, cholesteryl esters (CEs) reside in hydrophobic cores of circulating lipoproteins and intracellular lipid droplets. However, the inert identity of CEs is dramatically changed if cholesterol is esterified to a polyunsaturated fatty acid and subjected to oxidative modification. Post-synthetic, or epilipidomic, oxidative modifications of CEs are mediated by specialized enzymes, chief among them are lipoxygenases, and by free radical oxidation. The complex repertoire of oxidized CE (OxCE) products exhibit various, context-dependent biological activities, surveyed in this review. Oxidized fatty acyl chains in OxCE can be hydrolyzed and re-esterified, thus seeding oxidized moieties into phospholipids (PLs), with OxPLs having different from OxCEs biological activities. Technological advances in mass spectrometry and the development of new anti-OxCE antibodies make it possible to validate the presence and quantify the levels of OxCEs in human atherosclerotic lesions and plasma. The article discusses the prospects of measuring OxCE levels in plasma as a novel biomarker assay to evaluate risk of developing cardiovascular disease and efficacy of treatment.

## Introduction

Cholesterol esterification is a mechanism the body uses to store and transfer cholesterol, while at the same time to avoid cellular toxicity of the excess of unesterified (often called free) cholesterol. However, oxidation of a cholesteryl ester (CE) drastically changes the part CE plays, from a subdued supporting actor to a contender for the leading role. The script, in other words, the specific physiological or pathological context, defines if oxidized CE (OxCE) plays a villain or the hero.

**Figure 1:**
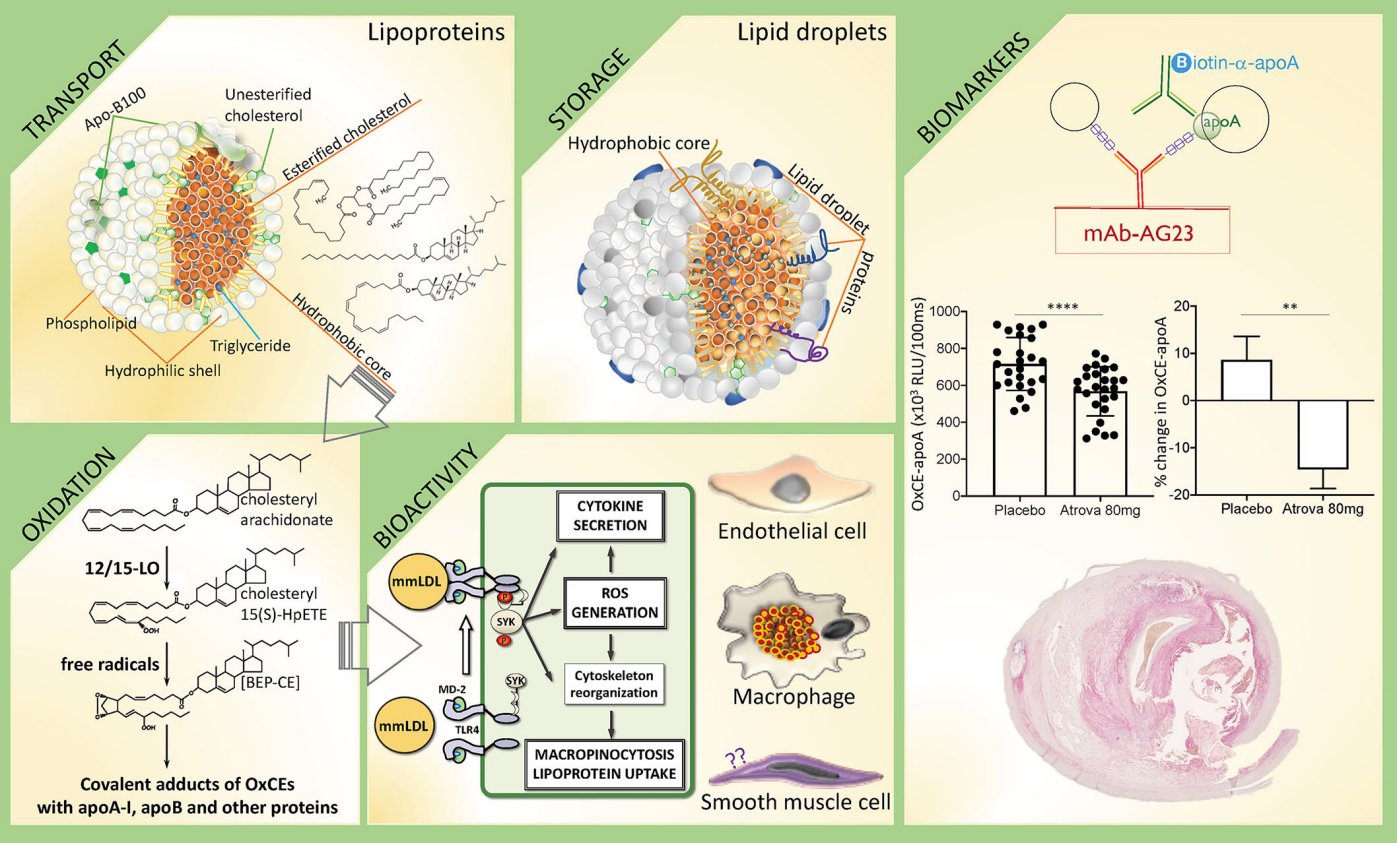
In search of CE identity—from inert storage to bioactivity and CVD biomarker. This diagram illustrates different biological processes that involve CEs, using examples described in text, and potential biomarker applications of detecting OxCEs in plasma and atherosclerotic plaques. **Transport:** Cholesteryl esters (CEs) together with triglycerides (TGs) populate the hydrophobic core of circulating lipoproteins, serving to deliver cholesterol and fatty acids to organs. Depicted is the low-density lipoprotein (LDL), a major CE-transporting lipoprotein in blood. Shown are representative structures (from top to bottom) of a TG, a CE with saturated fatty acyl [cholesteryl palmitate], and a CE with polyunsaturated fatty acyl (PUFA) [cholesteryl arachidonate], the latter is susceptible to oxidation. **Storage:** Intracellular lipid droplets predominantly store either CEs, like in macrophage foam cells in atherosclerotic lesions, or TGs, like in adipocytes. **Oxidation:** PUFA-CEs are the preferential substrate for 12/15-lipoxygenase (12/15-LO). Shown is cholesteryl 15(S)-HPETE, the product of 12/15LO-mediated oxidation of cholesteryl arachidonate, which in turn is oxidized to more complex products, like BEP-CE. Hydroperoxide, endoperoxide and aldehyde groups in OxCEs are reactive and can covalently modify proteins. **Bioactivity:** In one example of OxCE biological activity, BEP-CE and minimally modified LDL (mmLDL), which carry many OxCE molecules, activate an MD-2/TLR4/SYK pathway in macrophages, resulting in ROS generation, inflammatory cytokine secretion, and macropinocytosis-mediated LDL uptake and foam cell formation. OxCEs also activate endothelial cells, but their effects on vascular smooth muscle cells have not been studied. **Biomarkers:** Antibodies against OxCE-protein adducts stain human atherosclerotic lesions and recognize a fraction of ApoB and ApoA-I lipoproteins that carry OxCE in human plasma. In one example, an immunoassay measuring levels of OxCE-apoA lipoproteins detects reduced levels of this potential biomarker in subjects after treatment with atorvastatin compared to placebo. The artwork in this figure uses panels originally published in the Journal of Lipid Research. Gonen et al. A monoclonal antibody to assess oxidized cholesteryl esters associated with apoA-I and apoB-100 lipoproteins in human plasma. *J Lipid Res* 2019; 60:436-445. ^©^ The American Society for Biochemistry and Molecular Biology.

Unesterified cholesterol is an essential component of cellular membranes, where it plays both structural and signaling roles, the latter *via* regulation of lipid rafts and binding to many transmembrane proteins. In the nervous system, cholesterol is a major component of myelinated sheath of many nerve fibers. Cholesterol is also a precursor for biosynthesis of steroid hormones and bile acids. Thus, no wonder that cholesterol homeostasis is under tight control to ensure proper cellular and systemic functions. Dysregulation of cholesterol metabolism underlies many pathologies, from cardiovascular disease (CVD) to neurodegenerative disorders to cancer (1, 2). In homeostatic state, cellular cholesterol content is tightly controlled by balancing *de novo* synthesis, uptake of lipoproteins, export to extracellular milieu, and storage (2–4). The strategy for storage and transport of amphipathic cholesterol molecules is their esterification to fatty acids and tight packaging of the resulting hydrophobic CEs in the core of intracellular lipid droplets or circulating lipoproteins (**Figure 1**—Transport and Storage).

Inside the cells, after a threshold level in cellular cholesterol mass has been reached, excess cholesterol is esterified in the ER by the enzyme acyl CoA cholesterol acyltransferase (ACAT), and the newly synthesized CEs are stored in lipid droplets. To re-enter the cellular pathway, CE is hydrolyzed by neutral CE hydrolase (NCEH). Alternatively, lipid droplets are packaged into autophagosomes and fuse with lysosomes, where the CE is hydrolyzed by lysosomal acid lipase (LAL), generating unesterified cholesterol for delivery to cellular membranes or for export (5). Cellular CEs undergo a continual cycle of hydrolysis and re-esterification with a half-life of about 24 h (6, 7).

In circulation, lower density lipoproteins transport CEs from digestive organs to tissues, and high-density lipoprotein (HDL) returns excess cholesterol in the form of CE back to the liver. In brief, very-low-density lipoprotein (VLDL), packing triglycerides (TGs) and CEs, undergoes intravascular remodeling by shedding TGs and transitions into low-density lipoprotein (LDL), which is the most CE-rich lipoprotein in circulation. LDL is internalized by many cell types and thus delivers CEs to tissues. Serving the opposite function, HDL gathers excess of unesterified cholesterol from extrahepatic tissues in order to move it back to the liver. The HDL-associated enzyme lecithin-cholesterol acyltransferase (LCAT) catalyzes the esterification of free cholesterol with a fatty acyl transferred from the *sn-2* position of phosphatidylcholine (PC), resulting in the formation of a CE. In addition, there is a bidirectional exchange of CEs and TGs between HDL and the apoB-containing lipoproteins VLDL and LDL, mediated by the CE transfer protein (CETP) in plasma (8, 9). In plasma of healthy human subjects, approximately 70% of cholesterol molecules are esterified and reside in lipoprotein cores (10, 11).

This abridged description of CE metabolism illustrates complex pathways involved in keeping CE locked away in hydrophobic cores of lipid droplets and lipoproteins for storage and safe passage through the body—that is until CE undergoes oxidation and becomes biologically active.

### CE Oxidation

The acyl chain in a CE can be derived from a saturated, monounsaturated or polyunsaturated fatty acid (PUFA). The most common PUFA-CEs are cholesteryl linoleate [CE(18:2)], arachidonate [CE(20:4)], and docosahexaenoate [CE(22:6)]. The PUFAs are more susceptible to oxidation than cholesterol due to the presence of a weaker C-H bond at the bis-allylic position and will therefore be oxidized preferentially (12). The hydrogen atoms are easily abstracted from the bis-allylic positions of PUFAs to form a lipid radical - the first intermediate of enzymatic or non-enzymatic lipid peroxidation (13, 14). This article will be largely focused on the OxCEs with oxidized acyl chain and non-oxidized sterol, with a brief discussion of oxysteryl-containing OxCEs.

The enzyme 12/15-lipoxygenase (mouse 12/15-LO is highly homologous to human and rabbit 15-LO) differs from other PUFA-oxidizing enzymes like cyclooxygenases in that its preferential substrate is a CE and not a free fatty acids or a phospholipid (PL) (12, 15). In a test-tube reaction of LDL oxidation by rabbit 15-LO, even when the LDL particle is loaded with free linoleic acid, cholesteryl linoleate constitutes the major 15-LO substrate (15). However *in vivo*, 12/15-LO is an intracellular enzyme and LDL is an extracellular lipoprotein, so it was at first puzzling how CEs were oxidized by 12/15-LO. According to one suggested mechanism (16, 17), LDL binds to macrophage LDL receptor related protein-1 (LRP-1), which in turn induces 12/15-LO translocation from the cytosol to the cell membrane and mediates CE transport from LDL to the cell membrane, where it becomes oxidized by 12/15-LO, as well as the return of an OxCE to the LDL. It is unknown if any of lipid droplet-associated proteins can mediate a similar CE exchange and if CE oxidation can occur on the surface of intracellular lipid droplets.

Products of 12/15-LO–mediated CE oxidation are vulnerable to subsequent oxidation in free radical reactions, forming numerous and complex isoprostane OxCE products, with up to 6 oxygen atoms inserted in the molecule of cholesteryl arachidonate (18, 19). Among these polyoxygenated CE products, molecules with a bicyclic endoperoxide group (18–20), such as cholesteryl (9,11)-epidioxy-15-hydroperoxy-(5*Z*,13*E*)-prostadienoate (abbreviated as BEP-CE for the presence of bicyclic endoperoxide and hydroperoxide groups), have biological activities and can covalently modify proteins, including apolipoproteins, as discussed below. OxCEs can also decompose to produce highly reactive end products, like malondialdehyde (MDA) or 4-hydroxy-2-nonenal (4-HNE), which in turn covalently modify proteins and phosphatidylethanolamines (21, 22). These posttranslational modifications profoundly affect protein function.

In addition, intracellular OxCE hydrolysis and subsequent re-esterification of an oxidized fatty acyl chain can produce oxidized PL (OxPL) in the cell (12). In wild type but not 12/15-LO–deficient murine macrophages, radioisotope-labeled cholesteryl linoleate and cholesteryl arachidonate, either intracellular or as part of LDL, were oxidized by the macrophage 12/15-LO, and the oxidized fatty acyls in OxPL molecules originated from the OxCEs (12).

### Esterification of Oxysterols

Oxysterols, derived from either enzymatic or non-enzymatic oxidation of cholesterol, are bioactive and play important regulatory roles (23, 24). Similar to esterification of cholesterol, esterification of oxysterols is mediated by ACAT in cells and LCAT in plasma, as well as by lysosomal phospholipase A2 (25, 26). Esterification of oxysterols in plasma shifts their distribution away from albumin to LDL and HDL (27), where approximately 95% of plasma esterified oxysterols are found (28–31).

### OxCE Trafficking

Transport of OxCEs in circulation and their uptake by cells occur *via* the same pathways that traffic non-oxidized CEs. HDL carries 85% of total plasma CE hydroperoxides. While HDL and LDL carry approximately equal numbers of CE hydroperoxide molecules per particle, the CEs in HDL on a per lipid basis are over 20-fold “more oxidized” than those in LDL (32). Lipid peroxidation products in HDL are increased due to direct oxidation, transfer from LDL to HDL or by enzymatic re-esterification from OxPL by LCAT (9, 33, 34). CETP does not distinguish between CE and OxCE and mediates exchange of OxCE between HDL and LDL at the same rate as it transfers CE (9). Oxidized cholesteryl linoleate in which the fatty acyl moiety is oxidized to a hydroperoxide moves readily from HDL to hepatoma cells in serum-free medium (35), and the rate of SR-BI-mediated OxCE uptake by cells is approximately 9 times faster than that of non-oxidized CE and at least 40 times faster than the uptake of a whole HDL lipoprotein (36, 37). Because of the presence of hydrophilic, oxygen-containing groups in OxCE molecules, they become amphipathic and more mobile and presumably less confined to hydrophobic cores of lipoproteins or lipid droplets.

### Biological Activity of OxCE

There is an important feature of OxCEs that sets them apart from other oxidized lipids—a combination of reactive and/or functional oxidation moieties in the fatty acyl chain, which we already discussed, with cholesterol. Unmodified cholesterol is a major regulatory molecule for many proteins. The cholesterol recognition/interaction amino acid consensus (CRAC) motif and its reverse version CARC are present in many transmembrane proteins and are essential for their function (38). In model binding experiments, cholesterol is often replaced with an acidic short-chain CE, cholesteryl hemisuccinate (39), suggesting that cholesterol esterification does not significantly affect its interaction with CRAC/CARC motifs. However, we have not seen direct experimental or modeling comparison of cholesterol and long-chain CE or OxCE binding to these domains. And we are unaware of studies of interaction between transmembrane proteins, like GPCRs, with OxCEs, which are bifunctional—carrying both unmodified cholesteryl and fatty acyl oxidation moieties.

In addition to transmembrane cholesterol-binding proteins, there are non-membrane proteins that have hydrophobic pockets where cholesterol docks, CETP and Niemann-Pick disease, type C2 protein (NPC2) being the most characterized proteins in this class. Another cholesterol-binding protein is MD-2 (40). MD-2 is the LPS-binding co-receptor for TLR4, an obligatory component for LPS-induced TLR4 activation and signaling. MD-2 has a β-cup fold structure composed of two antiparallel β sheets forming a hydrophobic pocket, with positively charged residues located near the opening rim of the pocket (41–43). Fatty acyl chains of LPS dock into the hydrophobic pocket, and negatively charged phosphate groups of LPS bind positively charged residues at the pocket opening. Likewise, in the molecule of cholesterol, a hydrocarbon chain together with the steroid form an elongated hydrophobic structure, which docks in the hydrophobic pocket of MD-2, and a hydroxyl group linked to the other side of the steroid stabilizes cholesterol at the positively charged entrance to the pocket. Test-tube experiments confirm that MD-2 binds cholesterol (40). Furthermore, cholesterol is found associated with the MD-2 immunoprecipitated from human plasma or from mouse atherosclerotic lesions (40). It is unlikely that unesterified cholesterol binding to MD-2 activates TLR4 because there is no moiety in the MD-2-bound cholesterol that would interact with TLR4, however, such a moiety is present in the cholesteryl esterified to a fatty acyl–CE. The hypothesis that an oxygenated fatty acyl chain in OxCE provides additional interaction surfaces, which, in combination with cholesteryl anchoring in the MD-2 hydrophobic pocket, provide sufficient interfaces for OxCE-induced MD-2-TLR4 binding, remains untested.

Although structural determinants are not yet elucidated, OxCE, and specifically BEP-CE, indeed induces MD-2 recruitment to TLR4 and TLR4 dimerization (44). Interestingly, MyD88, a TLR4 adaptor which mediates the bulk of LPS effects, minimally contributes to macrophage responses to minimally modified LDL (mmLDL), a major carrier of OxCE. Instead, spleen tyrosine kinase (SYK) has been identified as a kinase, which is recruited to TLR4 and mediates the majority of mmLDL- and OxCE-induced effects in macrophages (45–47). This dichotomy between LPS- and OxCE-mediated TLR4 responses attests, in addition to the pattern-recognition character of TLR4, to the TLR4 functional selectivity, similar to biased agonism of GPCRs (48).

The SYK-dependent activation of TLR4 by mmLDL and OxCE results in profound cytoskeleton changes in macrophages, including actin polymerization, cell spreading, membrane ruffling and macropinocytosis (44, 46, 49) (**Figure 1**—Bioactivity). Macropinocytosis is a robust mechanism of OxLDL, mmLDL, and native LDL uptake by macrophages and foam cell formation. In addition, mmLDL induces PLCγ, PKC and NOX2-dependent ROS production, which regulates expression of RANTES (CCL5), IL-1β, and IL-6 (45). NOX-2 also regulates mmLDL-induced expression of MCP-1 (CCL2), TNFα, MIP-2 (CXCL2), and MIP-1α (CCL3) (45). *Tlr4^−/−^* primary macrophages fail to respond to mmLDL or OxCE (44, 46, 49). Remarkably, in *in vitro* experiments, mmLDL and low-dose LPS, imitating subclinical endotoxemia observed in patients with the metabolic syndrome, synergize to produce higher levels of inflammatory cytokines. Although published data point to pro-inflammatory effects of OxCE, a more extensive literature on biological effects of OxPL describes both pro-and anti-inflammatory effects depending on the disease or pathological condition context (21). Thus, we cannot exclude the possibility of context-dependent, anti-inflammatory effects of OxCE, but this requires further research.

In contrast to the biological activity of fatty acyl-oxidized OxCE, esterification of oxysterols largely serves to curtail their biological activity (50). However, in neurons, ACAT-mediated esterification of 24(S)-hydroxycholesterol results in the formation of atypical lipid droplets and neurotoxicity (51). These findings suggest cell type and context dependent effects of esterification of oxysterols.

In addition to free lipid OxCE, cholesteryl fatty acyl hydroperoxides or endoperoxides (like in BEP-CE) can make covalent adducts with proteins and thus affect their function and/or produce novel protein-OxCE epitopes. For example, cholesteryl hydroperoxyoctadecadienoate (HPODE) forms covalent adducts with PDGF, TGFβ, and bFGF and inactivates them (52). In contrast, cholesteryl HPODE does not modify EGF (52), implying specificity of OxCE-protein modification, however, determinants of this specificity remain unclear. Similarly, remain unclear the exact mechanisms of cholesteryl HPODE and cholesteryl 9-oxononanoate (9-ON) induced activation of PKC and ERK1/2 in endothelial cells, which results in expression of fibronectin connecting segment-1 and enhanced adhesion of monocytes to endothelial cells (53). Cholesteryl 9-ON induces expression of both TFG-β and TGF-β receptor type I in human U937 promonocytic cells. This effect is mediated by ERK1/2 and potentially is involved in sustaining vascular remodeling in atherosclerosis (54). Cholesteryl HPODE, but not HPODE-containing OxPL, has been identified as an active component that induces PPARα-dependent expression of CD36 in human monocyte-derived macrophages (55). It is also possible that in the experimental systems employed in the above experiments, cholesteryl HPODE underwent further oxidative modifications, resulting in more complex products, which in turn evoked biological activity different from that of an initial hydroperoxide.

### OxCE in Human Atherosclerotic Lesions

How relevant is the biological activity of OxCE to the pathogenesis of atherosclerosis? Definitive studies for OxCE are yet to be conducted but if any guide, recent work from Witztum’s group has demonstrated that constitutive expression of the single-chain E06 antibody, which neutralizes atherogenic effects of OxPL, in *Ldlr^−/−^* mice significantly reduces atherosclerosis and its co-morbidities (56, 57). In the absence of similar work targeting OxCE, we can only hypothesize that the substantial presence of OxCE in atherosclerotic lesions may have an atherogenic effect. Indeed, using advanced mass spectrometry techniques helped identify many OxCE species in human atherosclerotic lesions, estimating that, on average, 23% of cholesteryl linoleate, 16% of cholesteryl arachidonate and 12% of cholesteryl docosahexaenoate are oxidized (58). In a different study, OxCEs have accounted for 11 to 92% of the CE-PUFA pool in atherosclerotic plaques from different subjects (59). BEP-CE has been detected in human atherosclerotic lesions as well (44).

The studies cited in the previous paragraph identified free lipid OxCEs. It is technically challenging to construct a mass spectrometry method that would detect covalent OxCE adducts to proteins. However, these new epitopes can be detected with antibodies raised against the OxCE moiety independent of a protein, which has been covalently modified by this OxCE. For example, a monoclonal antibody raised against proteins modified with cholesteryl 9-ON has been shown to stain atherosclerotic lesions (60). Studies in our lab have produced a new monoclonal antibody that recognizes an OxCE epitope on modified proteins. To ensure the independence of an OxCE epitope recognition from the protein carrier, mice were immunized with OxCE-KLH and the antibody was selected against OxCE-BSA. The resulting antibody, AG23, bound OxCE-modified KLH, BSA, apoA-I, and a 6-amino acid peptide (61). The OxCE used for covalent modification of these proteins was a product of cholesteryl arachidonate oxidation with 2,2’-azobis (2,4-dimethylvaleronitrile) in an atmosphere of oxygen, which predominantly produced BEP-CE, but other complex oxidation products were present as well (44). The AG23 immunoreactivity was abundant in human carotid endarterectomy specimens, demonstrating the presence of OxCE epitopes in atherosclerotic lesions (**Figure 1**—Biomarkers) and suggesting relevance of OxCE to the pathogenesis of human CVD (61).

### OxCE in Human Plasma as a Biomarker for CVD

As with detection of OxCE in atherosclerotic tissue, early studies employing biochemical and mass spectrometry techniques reported a wide range of CE hydroperoxides in human plasma, from 3 to 920 nmol/L (62, 63), with hydroperoxides of cholesteryl linoleate and cholesteryl arachidonate as the major oxidation products (32, 64–66). Plasma levels of CE hydroperoxides have been significantly increased on day 1 and peaked at day 5 after subarachnoid hemorrhage, returning to normal levels on days 7 and 9 (67). This temporal sequence correlates well with the known time course of cerebral vasospasm, which typically has its onset between 5 and 7 days after subarachnoid hemorrhage. Using a targeted lipidomic approach to quantify multiple classes of OxCE, Yin’s group tested plasma samples from 49 CVD patients and observed a significant elevation of multiple oxidation products of cholesteryl arachidonate and cholesteryl linoleate in plasma of patients with myocardial infarction compared to that of control and other CVD groups. These results suggest release of OxCEs from the raptured atherosclerotic plaque (68).

The AG23 mAb against OxCE described in the preceding section has been used to develop a new ELISA method to measure OxCE associated with apoA-I or apoB-100 lipoproteins in human plasma (61). This assay measures levels of lipoproteins that have at least one OxCE epitope. Measuring OxCE-apoB and OxCE-apoA in plasma samples from PROXI, a randomized parallel-arm double-blind placebo-controlled trial in which human subjects received placebo or a statin treatment for 16 weeks, we demonstrated that the OxCE-apoA levels were significantly lower in subjects treated with atorvastatin than in the placebo group, independent of the apoA-I levels (**Figure 1**—Biomarkers) (56). Testing larger cohorts of human subjects with different conditions and treatment regimens will determine if this particular OxCE assay will become a useful diagnostic and/or prognostic CVD biomarker.

## Conclusions and Unresolved Questions

Biological activity and biomarker potential of OxCEs remain understudied. In part, this is due to a common perception of CEs as inherently inert intracellular “storage” and lipoprotein “transport” lipids, which is certainly the case for CEs with saturated fatty acyls. However, there exist mechanisms for oxidative modification of CEs with polyunsaturated fatty acyls, producing a multitude of OxCEs, which exhibit biological activity as free lipid and can covalently modify proteins. The unique feature of OxCEs is that they contain both an oxidized fatty acyl, which is often reactive and/or makes OxCEs less hydrophobic and thus more mobile, and the unmodified cholesteryl, which binds to and modulates function of many membrane and soluble proteins. In this article, we reviewed how cholesterol binding to MD-2 makes OxCE an agonist for TLR4, resulting in inflammatory and lipid accumulation responses in macrophages. Future studies will elucidate whether OxCE can interact with GPCRs, which potentially may have broad implications. More work is also needed to understand biological effects of esterified oxysterols, which seem to be tissue and pathology context dependent. The spectrum of proteins in plasma and in tissues that have OxCE covalent adducts remains unexplored, as remain poorly understood determinants of the OxCE specificity in covalent modification of proteins. The development of new antibodies that recognize OxCE-protein covalent complexes independent of a protein carrier will be instrumental in answering these questions. However, there remains a challenge of characterization of the exact chemical structure of OxCE-protein covalent adducts; where mass spectrometry methods are insufficient, co-crystallization of antibodies with their OxCE antigens may become a productive approach. Initial studies using OxCE-specific antibodies, as illustrated in this article, are promising but examination of larger cohorts of subjects with CVD and possibly other conditions are needed to fully evaluate the diagnostic and prognostic potential of OxCE as a new biomarker.

## Author Contributions

AG and YM wrote this manuscript. All authors contributed to the article and approved the submitted version.

## Funding

Work in authors’ laboratory is supported by the National Institute of Health Grants HL135737 and HL136275.

## Conflict of Interest

AG and YM are inventors listed on patents and patent applications related to the topic of this article. YM is a scientific co-founder of Raft Pharmaceuticals LLC. The terms of this arrangement have been reviewed and approved by the University of California, San Diego, in accordance with its conflict of interest policies.
